# Evaluation of the use of anxiolytics among students from Gabes Institute of Nursing Sciences

**DOI:** 10.1192/j.eurpsy.2025.958

**Published:** 2025-08-26

**Authors:** S. Kolsi, W. Abbes, S. Nouili, A. Bouaziz, K. Medhaffar

**Affiliations:** 1 Psychiatry, University Hospital, Gabes, Tunisia

## Abstract

**Introduction:**

The use of anxiolytics is a complex phenomenon, increasingly affecting the student population. Inappropriate use of this type of treatment can lead to abuse and even dependence.

**Objectives:**

To study the prevalence of dependence on anxiolytics, particularly benzodiazepines BZD in this population and to identify its associated factors.

**Methods:**

This was a cross-sectional descriptive study carried out among students from Gabes institute of nursing sciences, for a period of two months(March to May 2024).

Data were collected using an online anonymous questionnaire from Google form that we distributed via Messenger social network.

We used the Benzodiazepine Attachment Cognitive Scale (BACS) to study BZD dependence.

A score ≥ 6 enables to differentiate between dependent and non-dependent patients.

**Results:**

We collected data from 135 students. Our sample included only 33 students.

The mean age of our population was 21.30 ±1.51 years and the sex ratio (M/F) was 0.65.

Consumption of psychoactive substances (PAS) was reported by 21 students (63.63%): tobacco by 21.2%, coffee by 12.2% and alcohol by 12.2%.

All students reported having taken benzodiazepines BZD.

In our study, 13 students (39.4%)reported having used BZDs 1 or 2 times in their lives (for experimental purposes). Daily use of BZDs was not reported.

The mean score of the ECAB scale was 6.93, with extremes of[3-10].

According to our results, dependence on BZD was clearly predominant, found in 28 students (84.8%). No correlation was found between the socio-demographic characteristics and the presence of anxiolytic dependence.

BZD dependence (assessed by the ECAB scale) was correlated with coffee consumption (p=0.03), unlike for other substances (tobacco, alcohol, cannabis).

**Conclusions:**

The study highlighted the high level of dependence on anxiolytics, particularly BZD and identified individuals who were at higher risk. Specific interventions were necessary to deal with the problem of addiction.

**Disclosure of Interest:**

None Declared

